# Probing and Interpreting the Porosity and Tortuosity Evolution of Li-O_2_ Cathodes on Discharge through a Combined Experimental and Theoretical Approach

**DOI:** 10.1021/acs.jpcc.0c10417

**Published:** 2021-02-25

**Authors:** Amangeldi Torayev, Simon Engelke, Zeliang Su, Lauren E. Marbella, Vincent De Andrade, Arnaud Demortière, Pieter C. M. M. Magusin, Céline Merlet, Alejandro A. Franco, Clare P. Grey

**Affiliations:** † Laboratoire de Réactivité et Chimie des Solides (LRCS), UMR CNRS 7314, Université de Picardie Jules Verne, Hub de l’Energie, 15 Rue Baudelocque, Amiens 80039, France; ‡ Department of Chemistry, University of Cambridge, Lensfield Road, Cambridge CB2 1EW, U.K.; § ALISTORE-European Research Institute, FR CNRS 3104, Hub de l’Energie, 15 Rue Baudelocque, Amiens 80039, France; ∥ Cambridge Graphene Centre, University of Cambridge, 9 JJ Thomson Avenue, Cambridge CB3 0FA, U.K.; ⊥ Réseau sur le Stockage Electrochimique de l’Energie (RS2E), FR CNRS 3459, Hub de l’Energie, 15 Rue Baudelocque, Amiens 80039, France; # Department of Chemical Engineering, Columbia University, 500 W 120th St, New York, New York 10027, United States; ∇ X-Ray Science Division, Advanced Photon Source, Argonne National Laboratory, Lemont 60439, United States; ○ CIRIMAT, Université de Toulouse, CNRS, Bât. CIRIMAT, 118, route de Narbonne, Toulouse cedex 9 31062, France; ◆ Institut Universitaire de France, 103 Boulevard Saint-Michel, Paris 75005, France

## Abstract

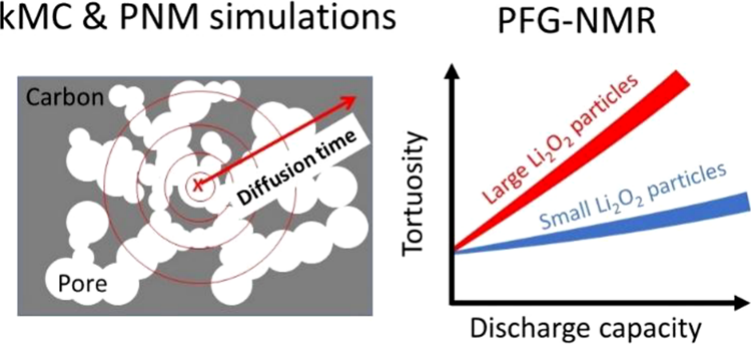

Li-O_2_ batteries offer a high theoretical discharge capacity due to the formation of light discharged species such as Li_2_O_2_, which fill the porous positive electrode. However, in practice, it is challenging to reach the theoretical capacity and completely utilize the full electrode pore volume during discharge. With the formation of discharge products, the porous medium evolves, and the porosity and tortuosity factor of the positive electrode are altered through shrinkage and clogging of pores. A pore shrinks as solid discharge products accumulate, the pore clogging when it is filled (or when access is blocked). In this study, we investigate the structural evolution of the positive electrode through a combination of experimental and computational techniques. Pulsed field gradient nuclear magnetic resonance results show that the electrode tortuosity factor changes much faster than suggested by the Bruggeman relation (an equation that empirically links the tortuosity factor to the porosity) and that the electrolyte solvent affects the tortuosity factor evolution. The latter is ascribed to the different abilities of solvents to dissolve reaction intermediates, which leads to different discharge product particle sizes: on discharging using 0.5 M LiTFSI in dimethoxyethane, the tortuosity factor increases much faster than for discharging in 0.5 M LiTFSI in tetraglyme. The correlation between a discharge product size and tortuosity factor is studied using a pore network model, which shows that larger discharge products generate more pore clogging. The Knudsen diffusion effect, where collisions of diffusing molecules with pore walls reduce the effective diffusion coefficients, is investigated using a kinetic Monte Carlo model and is found to have an insignificant impact on the effective diffusion coefficient for molecules in pores with diameters above 5 nm, *i.e.*, most of the pores present in the materials investigated here. As a consequence, pore clogging is thought to be the main origin of tortuosity factor evolution.

## Introduction

Lithium oxygen (Li-O_2_) batteries are potentially game-changing energy storage systems with a very high theoretical capacity,^1^ but they face a wide spectrum of very significant material challenges that need to be overcome before achieving commercialization.^2^ A Li-O_2_ battery consists of a lithium-containing anode (typically Li metal), a separator soaked with an ionically conducting but electronically insulating electrolyte-containing lithium ions (Li^+^), and a porous electronically conducting cathode (typically a porous carbon) in contact with oxygen gas. During discharge, lithium is oxidized at the anode and lithium ions migrate to the cathode where they react with oxygen (dissolved in the electrolyte) to form solid discharge products, predominantly Li_2_O_2_ (lithium peroxide). During charge, Li_2_O_2_ discharge products decompose and form Li^+^ and O_2_. The lithium ions migrate back to the anode where they are reduced. Oxygen molecules are released back to the oxygen gas source. The predominant discharge product, Li_2_O_2_, is an electronic insulator.^3^ During discharge, Li^+^ reacts with O_2_ to form LiO_2_ (lithium superoxide) close to the cathode surface. LiO_2_ can then form Li_2_O_2_ via further electrochemical reduction or through disproportionation. The growth of Li_2_O_2_ can be further divided into two processes.^4^ If the intermediate species, LiO_2_, is insoluble in the electrolyte, it is either directly reduced or it disproportionates to form a film of Li_2_O_2_ on the cathode surface. The thickness of this film is limited by the electron tunneling through Li_2_O_2_ leading to thin films of about 10 nm.^5^ If the solubility of LiO_2_ in the electrolyte is high, LiO_2_ molecules can diffuse away from the carbon surface forming large, toroidal-shaped discharge products via a disproportionation mechanism, which leads to pore clogging and impedes further transport of mobile molecules and ions in the electrolyte.^6−8^

In a Li-O_2_ cell, even though the active cathode (or positive electrode) material is oxygen, additional material is used to provide electronic conductivity and mechanical support to host the Li_2_O_2_ discharge products. This support structure is typically a porous carbon and is also designated as being a part of the cathode, although in principle it is not electrochemically active. The choice of carbonaceous cathode material and its structure has a large impact on the performance of the cell. There are two main capacity-limiting processes associated with the carbon cathode mesostructure. The first one is the passivation of the carbon surface area with electronically insulating discharge products,^9−12^ and the second one is the slow transport of reactants, particularly oxygen, through the porous network.^13−16^

The passivation issue has been demonstrated at the experimental level in several studies as follows: Laoire *et al.*(9) studied it through cyclic voltammetry and rotating disc electrode characterization and Albertus *et al.*(10) through a combination of experimental and modeling techniques. One approach to mitigate passivation is to use a cathode with an extremely high surface area such as graphene-based^17,18^ or nanostructured electrodes.^19^ Another strategy is to avoid the formation of passivating thin films by preferentially forming large discharge products through dissolution of the intermediate species. Selecting electrolyte solvents with high donor numbers^20−23^ or choosing specific salt anions^24^ can enhance the solubility of these intermediate discharge products and lead to large discharge product particles. Adding a small amount of water^25,26^ or redox mediators^27−29^ can also lead to the formation of large discharge product particles. While all of these methods reduce surface passivation, the formation of large discharge product particles often clogs the cathode pores, introducing new issues for reactant transport during electrochemical charge and discharge.

Transport limitations arise from a combination of the slow diffusion of oxygen and Li^+^ in the electrolyte and the tortuous nature of the porous cathode. Using electrolytes with high oxygen diffusion coefficients,^30,31^ and designing cathodes with high porous volume, and short and numerous diffusion pathways^32−34^ can help in obtaining better battery performance. Regardless of the type of cathode mesostructure selected, the porous medium changes along discharge due to the formation of solid discharge products: some pores get smaller and some pores get clogged, impeding the transport processes^13−16^ and motivating studies to investigate how the porous media change along discharge.

Bardenhagen *et al.*(14) investigated pore clogging for three gas diffusion electrodes along discharge using a three-electrode electrochemical impedance spectroscopy setup. They identified four processes with different time constants, which they attributed to lithium ion migration through a surface layer, charge-transfer from the carbon to the molecular oxygen, lithium and oxygen ion diffusion toward the cathode surface, and lithium ion movement inside the pores. Their observations confirmed that the pore clogging is a key limiting factor. In addition, they studied xerogel electrodes and showed that they provide high capacities due to the large volume of mesopores allowing for an improved oxygen transport.

To improve the battery performance, studies that directly investigate the transport properties, particularly tortuosity factor of the electrodes, are needed. Here, we present a work that focuses on the tortuosity factor evolution along discharge and investigate how it is affected by the type of electrolyte used. For this purpose, pulsed field gradient nuclear magnetic resonance (PFG-NMR) experiments are used. PFG-NMR spectroscopy represents a powerful tool to measure diffusion of mobile species in porous media, which has been used to study ion transport in batteries and supercapacitors.^35,36^ Forse *et al.* studied the effect of the pore size distribution on the self-diffusion of ions and measured diffusion in and out of the nanopores in YP50F and YP80F, two porous carbon materials used in supercapacitors. ^1^H, ^19^F, and ^13^C NMR experiments were conducted.^35^ It was shown that the diffusion of ionic species is more than two orders of magnitude slower in nanoporous materials compared to diffusion in the bulk electrolyte. The ionic concentration, which changes along charge and discharge in the supercapacitor systems, also influences diffusion. Engelke *et al.*(36) used ^1^H and ^7^Li PFG-NMR to measure anisotropic diffusion in model porous silicon substrates. They measured effective diffusion coefficients with incremental diffusion times, which allowed them to calculate the related mean square displacements and extract information about the pore dimensions. Stallmach *et al.*(37) and Kondrashova *et al.*(38) studied anisotropic self-diffusion in nanoporous materials to analyze orientation-dependent diffusivities, monitoring and quantifying diffusion of probe molecules along one-dimensional channels.

In this work, we use ^1^H PFG-NMR to study the tortuosity factor evolution when discharging Super P cathodes in Li-O_2_ batteries. We focus on long-range diffusion and measure effective diffusion coefficients of tetraglyme molecules in cathodes at different states of discharge. The tortuosity factor is computed from the ratio between the measured effective diffusion coefficient in the porous medium and the bulk diffusion coefficient. To study the effect of the solvent, we conduct experiments with two different electrolytes consisting in LiTFSI dissolved in either tetraethylene glycol dimethyl ether (tetraglyme) or in dimethoxyethane (DME). The tortuosity factor measurements are compared to tomographic imaging experiments on the discharged samples. To help interpret the experimental observations, we investigate the effect of pore sizes, and pore clogging, on the tortuosity factor, using a kinetic Monte Carlo (kMC) model and pore network model (PNM), respectively, as described in previous publications.^39,40^

## Methodology

### Cell Components Preparation

#### Anode

A lithium metal anode is used in the eight cells of this study. The lithium anode is cut in a disk shape with a 0.73 mm thickness and 9 mm diameter. Considering that the density of lithium metal is 0.534 g cm^-3^, this negative electrode is sufficient to provide a capacity of about 95.8 mA h (7725 mA h g^–1^, normalized to cathode mass) per cell, which ensures that the anode is not a capacity limiting parameter, as none of the cells reached this high discharge capacity.

#### Separator

Two layers of Whatman glass fiber with a diameter of 14 mm are used in each cell. The total thickness of these two layers is about 1 mm. Two layers are used to avoid any short circuit in the cell assembly.

#### Electrolyte

Two different solvents are used, dimethoxyethane (DME, anhydrous 99.5%, Sigma-Aldrich) and tetraethylene glycol dimethyl ether (tetraglyme, 99%, Acros Organic). LiTFSI (lithium bis(trifluoromethanesulfonyl)imide, Sigma-Aldrich) was used as a salt. Before use, the salt was dried in a vacuum oven at 120 °C overnight and transferred into an argon glovebox.

#### Cathode

Self-standing mesoporous Super P carbon electrodes were prepared from a mixture of 70% (mass percentage) Super P carbon (TIMCAL) and a 30% PTFE (polytetrafluoroethylene) binder in ethanol. The mixture was agitated using magnetic stirring at 70 °C for about 2 h, and the resulting paste was rolled into an approximately 300 μm thick film. The film was cut into electrodes with diameters of 11 mm. After initial drying of the electrodes in a 50 °C oven under ambient air, they were further dried under vacuum at 120 °C for 12 h. The dried electrodes were taken into an argon gas glovebox without air exposure and then used to make batteries. The mass loading of the electrode is around 13.0 ± 0.4 mg cm^–2^ (around 9.1 mg cm^–2^ of carbon mass loading, 70 wt%). The discharge capacities reported in this article are normalized by the total electrode mass (12.4 mg: mass of binder + carbon).

### Electrochemical Testing and Sample Preparation for PFG-NMR and Tomography

**Figure 1:**
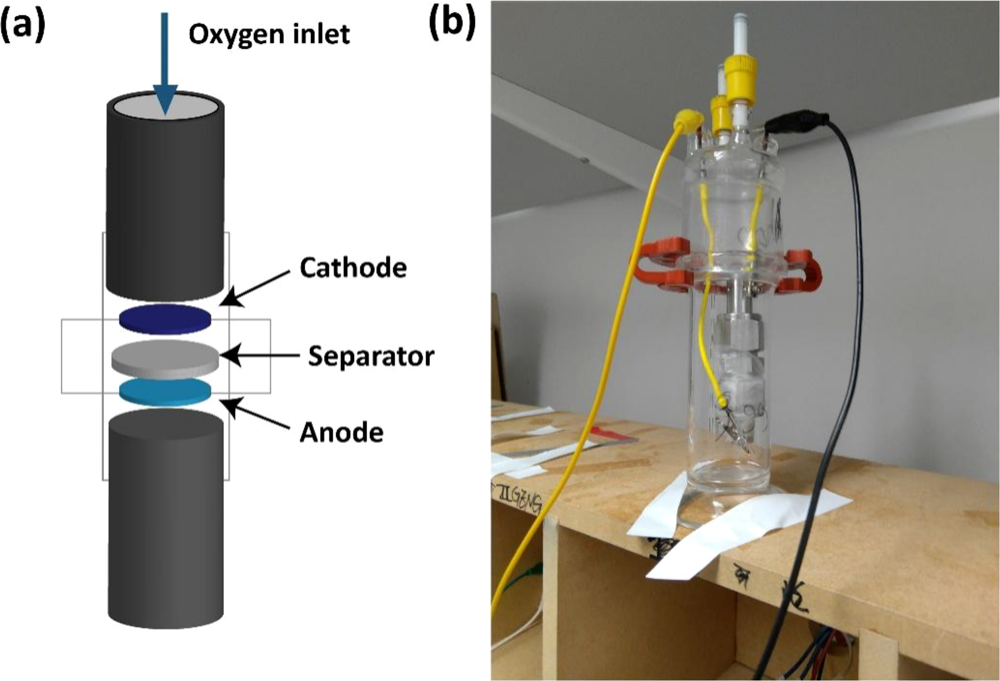
(a) Scheme of the Swagelok cell used for the electrochemical experiments. (b) The glass chamber used to keep the Swagelok cells under oxygen, which has space for a single Swagelok cell.

For the electrochemical experiments, Swagelok cells are used (Figure 1a). A hole was drilled through the plunger at the cathode side to connect to the oxygen supply. A lithium metal anode, two layers of separators, and a cathode were placed into the cell; then 250 μL of electrolytes was added. The cell was assembled in an argon glovebox and put into an airtight glass chamber (Figure 1b). Once the glass chamber was sealed, it was taken out of the glovebox and flushed with oxygen gas for 5 min to replace the argon gas with oxygen. After flushing and before the electrochemical tests, the cell was rested for 6 h to give a sufficient time for oxygen molecules to dissolve in the electrolyte.

The electrochemical tests have been carried out on a Bio-Logic VMP3 multichannel potentiostat. Four cells with 0.5 M LiTFSI in tetraglyme and four cells with 0.5 M LiTFSI in DME electrolytes were discharged galvanostatically with a current of 50 μA. The discharge process was stopped at different depths of discharge for the different cells, the Swagelok cells were disassembled, and the cathode mesostructures were analyzed by PFG-NMR (two cells discharged with tetraglyme and four cells discharged with DME electrolyte solvents) experiments and tomography (two cells discharged with the tetraglyme electrolyte solvent). When disassembling the cells, precautions were taken so that all measurements were conducted on samples that had not been exposed to air. After discharge, the cells were disassembled in a glovebox, and each cathode was put in a vial with pure DME to remove the salt and electrolyte. DME was used to wash out the salt because of its low viscosity and because it evaporates easily.

For the PFG measurements, the cathode was dried in the prechamber of the glovebox under vacuum. The dried electrode was then placed in another vial with pure tetraglyme. The vial with the tetraglyme-soaked electrode was put under vacuum for 3 min to remove gases inside the pores and help with the wetting of the small pores by the solvent molecules; the electrode was left to soak in the solvent for 1 h. After that, the electrode surface was gently dried on a lab paper-towel (as discussed below) and the electrode was put into a sealed plastic bag (Figure S1).

For the tomography experiments, the electrodes were transferred after washing with DME into an argon-filled glove-bag with an optical microscope for further sample preparation. The electrodes were chopped into small pieces. Thereafter beneath the microscope, one was selected, with the aid of a microcontroller and epoxy, a piece that fills the field of view (55 μm) of the transmission X-ray microscope. The mounted sample was inserted with care into a Kapton tubing and sealed by Torr Seal curing at 50 °C overnight to avoid humidity contamination of the Li_2_O_2_ particles.

### PFG-NMR Experiments

#### Pulse Sequence and Theory

**Figure 2:**
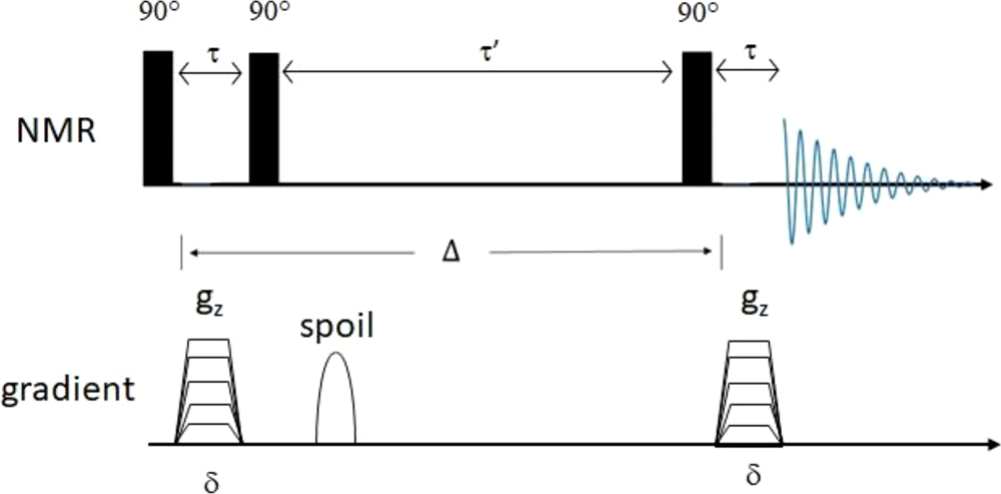
Pulse sequence used for the stimulated echo-pulsed field gradient NMR experiments: (top) NMR frequency pulses and (bottom) gradient pulses.

For diffusion NMR studies, the position of a nucleus is typically encoded by applying a linear magnetic-field gradient along the magnetic field axis, Δ*B*(*g_z_*) = *g_z_z*, superimposed on the static homogeneous magnetic field *B*_0_. In this work, the so-called STimulated-Echo (STE) pulse sequence 90°-τ–90°-τ′-90°-τ–acquisition combined with magnetic-field gradient pulses (Figure 2) was employed. The STE pulse sequence works well for NMR nuclei with transversal-relaxation times shorter than spin–lattice relaxation times, as is typically the case for molecules with slow or restricted mobility.

For a linear magnetic field gradient *g_z_* along the magnetic field axis, the total magnetic field experienced by the observed NMR nuclei is
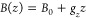
1After the initial 90° radio frequency (rf) pulse, a magnetic-field gradient pulse of duration δ is applied to encode the NMR coherences with the locations of the molecules in the system. The second 90° pulse at time τ stores this encoded coherence along the magnetic field axis. Then, the molecules are given a certain time τ′ to diffuse before the final 90° pulse followed by another gradient pulse (equal to the first) is applied to decode the location tags. The three rf pulses combined with the gradient pulses produce a stimulated-echo signal at time *t* after the last pulse.

The idea behind this pulse sequence is that if molecules do not move at all during Δ (the diffusion time between the two gradient pulses, approx. τ + τ′, Figure 2), the encoding and decoding magnetic fields will be the same and the echo intensity of the (stimulated) echo signal for these molecules will be maximal. By contrast, if molecules diffuse during the pulse sequence, then they will experience different magnetic encoding and decoding fields. Consequently, the intensity of the echo signal will decrease. An effective diffusion coefficient (*D*_eff_) can be determined by applying a range of gradients for a fixed value of Δ and subsequently fitting the signal intensity *I*′ as a function of gradient strength *g_z_* to the Stejskal–Tanner equation (see Figure S2).^41^ For the PFG stimulated echo experiment with the pulse sequence depicted in Figure 2, the equation is
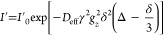
2where γ is the gyromagnetic ratio of the observed NMR nuclei, typically ^1^H.^41,42^ In practice, to avoid gradient-ringing effects, trapezoid-shaped gradient pulses were used. Any remaining coherence not stored along the magnetic field axis by the second 90° pulse was suppressed by the spoil pulse.

**Figure 3:**
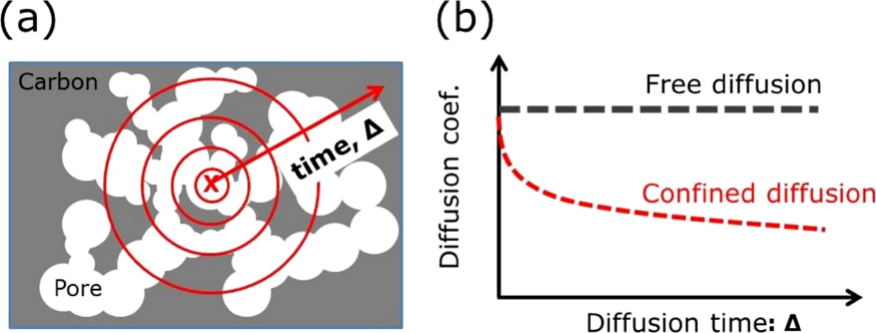
(a) Illustration of the area covered by a diffusing molecule with time. (b) Schematic representation of free diffusion and confined diffusion as a function of diffusion time.

For the pure tetraglyme measurement (bulk diffusion), the diffusion time Δ selected in the PFG-NMR experiment does not affect the diffusion coefficient as the diffusing molecules are not restricted in their motion. For the diffusion in porous media, the diffusion time plays an important role. If the diffusion time is too short, then, the diffusing molecules will not encounter any pore walls, and the calculated diffusion coefficient will be similar to the bulk diffusion coefficient. When increasing Δ, the mobile molecules will diffuse further and collide with pore walls (Figure 3a). Hence, the effective diffusion coefficient will decrease (Figure 3b). For sufficiently large diffusion times, the confined diffusion coefficient converges to a value corresponding to long range diffusion, which is the one suitable for extracting the tortuosity factor. The NMR technique used here is not spatially resolved, and in a Li-O_2_ battery, the discharge products can have non-uniform distribution along the electrode thickness due to slow oxygen transport. A low discharge current density has been used to minimize this effect.

#### Relation between the Tortuosity Factor and Diffusion Coefficients

The porosity of an electrode is the fraction of the void (pore) volume in the total electrode volume. The tortuosity factor is a measure of the geometric complexity of a porous medium, and it is usually defined as the squared ratio between the curved paths followed by a fluid in the porous medium over a straight path. The porosity and tortuosity factor both affect the effective diffusion coefficient in a porous electrode. When there is a more porous volume (high porosity), then, the diffusion is less restricted, so the porosity and effective diffusion coefficients are positively correlated with each other. When the tortuosity factor increases, the diffusion paths of the mobile species are more curved, and their effective diffusion coefficients decrease. Hence, the tortuosity factor and effective diffusion coefficients are anticorrelated. Following these intuitive considerations, the tortuosity factor (τ) is defined with respect to the effective diffusion coefficient (*D*_eff_), the bulk diffusion coefficient (*D*_0_) and the porosity (ε) as^43^
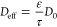
3By knowing *D*_0_ and *D*_eff_, one can calculate .

In this work, the porosity and tortuosity factor are considered to be a reflection of the structural properties of the carbon cathode. That is, they mainly depend on the porous medium and its mesostructure and not on the nature of the diffusing species, implying that the diffusing species do not bind to the carbon mesostructure, or that the nature of the interaction does not vary between different solvents. Different molecules or ions will not necessarily experience the same porosity and tortuosity factor in a given medium. For example, if molecule B is twice as big as molecule A, some parts of the porous electrode might not be accessible to B due to its size, and as a result, a smaller effective porosity and larger tortuosity factor will be determined when using B as a probe. The porosity and tortuosity factor might also depend on the rigidity/flexibility of molecules A and B. In practice, however, the size differences between relatively similar molecules, *e.g.* the different solvents used in Li-O_2_ batteries electrolytes, are limited, and for the purpose of the current work, it is possible to assume that the porosity and tortuosity factor measured with these molecules reflect conditions experienced by typical battery electrolytes inside the carbon structure. The measurements require a molecule with the appropriate diffusion coefficient and relaxation time to sample the different pores and move a sufficient distance within the timescale of the PFG-NMR experiment; here, the motion of ^1^H nuclei in the tetraglyme solvent was tracked by NMR. It is worth noting that oxygen molecules cannot be probed directly using ^17^O NMR due to the paramagnetic character of O_2_ and the low natural abundance of ^17^O. As the oxygen molecules are dissolved in the electrolyte, we assume that the effect of confinement on these molecules will be similar to the one on the solvent molecules.

#### Experimental Details

A 300 MHz Avance III Bruker NMR spectrometer equipped with a gradient amplifier, Diff50 gradient stack, and a 10 mm ^1^H coil configuration was used for the ^1^H PFG NMR experiments. 90° pulses of 14.7 μs, a (half-height) gradient duration δ of 1 ms, and a time τ between the first two pulses of 1.5 ms were employed. The overall diffusion time Δ ≈ τ + τ′ was varied between 10 and 1200 ms. The gradient amplitude *g_z_* was varied between 0 and 200 G cm^–1^ (calibrated versus the self-diffusion coefficient of neat water, 2.20 10^–9^ m^2^ s^–1^, at 25°C). The STE was recorded in the time domain for 16 different gradient values. PFG-NMR measurements were conducted as described above with a varying diffusion time. For the long-range effective diffusion coefficients, the PFG-NMR measurements with diffusion time 1200 ms are used. The NMR experiments are carried out at 25°C with temperature controlled with water flow.

The STE signals were then transformed into the frequency domain, the resulting NMR peaks integrated, and the peak integrals plotted as a function of the gradient value. NMR data processing is done within the Bruker NMR software Topspin to extract intensities. Further analysis and fitting of intensities to the Stejskal–Tanner equation are done with MATLAB software.

### Tomographic Imaging

An X-ray nano-tomography experiment with 54 nm spatial resolution (voxel size 54 nm) was done using 50 nm outermost zone width Fresnel zone plate optics and was performed in 32-ID-C in the advanced photon source (APS) synchrotron at Argonne National Laboratory.^44^ A Beam Shaping Condensor of a 60 nm outermost zone width was used. The in-line Zernike phase contrast technique involving a phase ring in the back focal plane of the zone plate was used to increase the contrast between carbon and lithium peroxide.^45^ Measurements were performed at 8 keV with a monochromatic beam (Δ*E*/*E* = 10^–4^). The distance sample to detector of 3329 mm provided an X-ray magnification of 46. Projections of 721 were acquired within 180° (0.25°/frame and 1 second per frame) rotation with a FLIR GS3-U3-51S5M-C detector. Projections were preprocessed with packages present in Tomopy^46^ and then reconstructed into volumes by the GPU-accelerated SIRT method^47^ (200 iterations, more iterations induce noises) in the same library. The WEKA trainable segmentation Fiji plug-in Random Forrest based machine learning was used for the multiphase segmentation.^48^ The surface, porosity, and tortuosity factor determinations are conducted respectively with in-house codes developed by the authors^39^ and Taufactor;^49^ Taufactor calculates the tortuosity factor from the tomographic images, simulating diffusion by solving Fick’s second law.^49^

### Pore Network Model

To assess the effect of the discharge product growth mechanism on pore clogging, a pore network model already described in previous publications was used.^39,40^ The galvanostatic discharge potential profiles (the discharge current density is 100 μA g^–1^) for a Super P pristine electrode mesostructure obtained via tomographic imaging were simulated.^39^ A tomographic image of the pristine Super P electrode from our previous work^39^ was used as we do not have the tomographic image for the pristine electrode used in this work. As the tomographic image in our previous work and the current study use the same Super P material from the same supplier, their microstructure should have a similar morphology. A system size of 4 × 4 × 5 μm, along two radial and the electrode thickness directions, respectively, was used. In the model, an overall electrochemical reaction is considered where two lithium ions react with an O_2_ molecule to form Li_2_O_2_. Five simulations were completed on the same starting electrode mesostructure with five different escape factors (χ = 0, 0.25, 0.5, 0.75, and 1) to study the effect of the discharge particle growth mechanism on the pore clogging and tortuosity factor evolution. The escape factor represents the propensity of LiO_2_ intermediate molecules of the discharge process to dissolve in the electrolyte and contribute to the growth of large discharge particles. By increasing the escape factor from 0 to 1, we move from a thin-film growth to a solution phase growth mechanism (and the formation of large particles).

### Kinetic Monte Carlo Model

To study the effect of pore size and the importance of the Knudsen diffusion effect in the systems explored here, we developed a kinetic Monte Carlo model. The Knudsen effect describes the diffusion in narrow pores where the diffusion coefficient is affected by collisions of the mobile species with the pore wall. While intuitively, smaller pores will lead to more collisions and slower diffusion, a model was constructed to obtain quantitative information on this effect for the pore sizes relevant to this study. The kMC model is adapted from a previously published model, which had been used to simulate the discharge process in Li-O_2_ batteries.^50^

**Figure 4:**
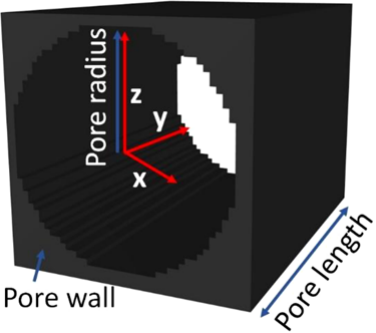
Cylindrical pore geometry considered in the kMC model.

A cylindrical pore geometry (Figure 4) was used to simulate diffusion in a pore. The pore volume was sliced into voxels with a side length of 0.5 nm, which is assumed to be the hydrodynamic size of a dissolved O_2_ molecule. The hopping frequency (γ*_i_*), also called the event frequency, was calculated as
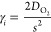
4where *D*_*O*_2__ is the bulk diffusion coefficient of O_2_, and *s* is the voxel size. In the model, the O_2_ molecules can jump (or diffuse) to 18 sites (6 face sharing and 12 edge sharing) provided that those neighboring voxels are empty. Periodic boundary conditions in the direction of the pore length were used.

In this kMC model, only translational motion and no reactions were simulated. A variable step size method is used^50^ and the time step is calculated as
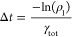
5
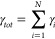
6where ρ_1_ is a random number in the interval (0,1], *i.e.*, larger than zero and smaller or equal to one, γ_tot_ is the sum of all event frequencies, and *N* is the total number of possible events.

The event occurring at a given step is chosen according to the weighted probability of each event. The event *j* is selected according to the following rule
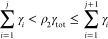
7where ρ_2_ is a random number in the interval (0,1]. This process allows for the simulation of a system with various event frequencies, including a mixture of fast and slow events. In the present case, only events related to the transport of a single type of molecule (O_2_) are considered. However, the script is written in a general form to be able to work with many event types if necessary.

It is worth noting that, in this work, an approach upgraded from the ones previously reported was used.^50,51^ A simplified scheme of our algorithm is given in Figure S3. In this new scheme, all possible events at the beginning of the calculation are determined and then stored in an easy-to-update data structure. In the main loop, only the events, which are in the vicinity of the executed events, are updated. The number of events updated at each timestep is around 18 against several thousands when the full ensemble of possible events is scanned. As a consequence, this procedure is much more efficient, and calculations are much faster: the new procedure is about 2000 times faster than the previously reported ones^50,51^ for the system under investigation here.

**Figure 5:**
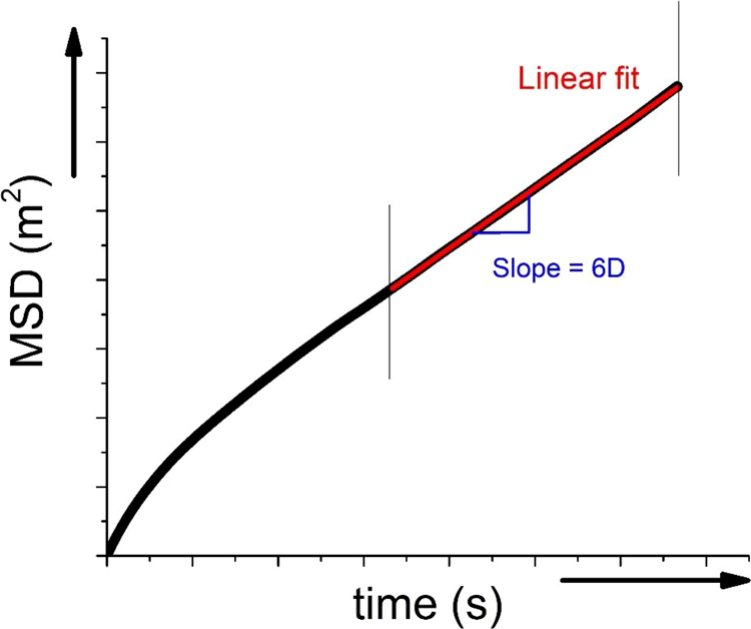
Illustration of the determination of a diffusion coefficient from an MSD curve. Two vertical lines show the time interval where the MSD curve is linear with time. The red line is the fit to the MSD curve in the selected region.

To extract the diffusion coefficients from the particles trajectories, the mean square displacement (MSD) method was used where the MSD function is defined as
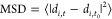
8where *d*_*i*, *t*_ is the position of molecule *i* at time *t* and *d*_*i*, *t*_0__ is the position of the same molecule at time *t* = 0. The ⟨.......⟩ sign corresponds to an average over all O_2_ molecules. After a long enough simulation time, the MSD curve becomes linear (Figure 5), and the diffusion coefficient (*D*) can be retrieved from its slope according to Einstein relation
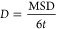
9

For kMC simulations with different cylinder diameters, if the cylinder length was kept the same for all pore sizes, there would be a very significant variation in the number of oxygen molecules in the pores as cylinders with large diameters would contain many more molecules than cylinders with smaller diameters. This would affect the MSD fitting accuracies. To have the same amount of oxygen molecules in all the calculations, the porous volume is kept constant and equal to 783,398 nm^3^ (*i.e.*, 6,267,184 voxels), which corresponds to the pore volume of a cylinder with a diameter of 100 nm and a length of 100 nm. This means that cylinders with smaller pore diameters will have larger pore lengths. The amount of oxygen in each calculation is set to 1256 molecules. The total simulation time is 2 μs.

## Results and Discussion

### Measurement of Bulk Diffusion Coefficients and Evaluation of Drying Procedures

**Figure 6:**
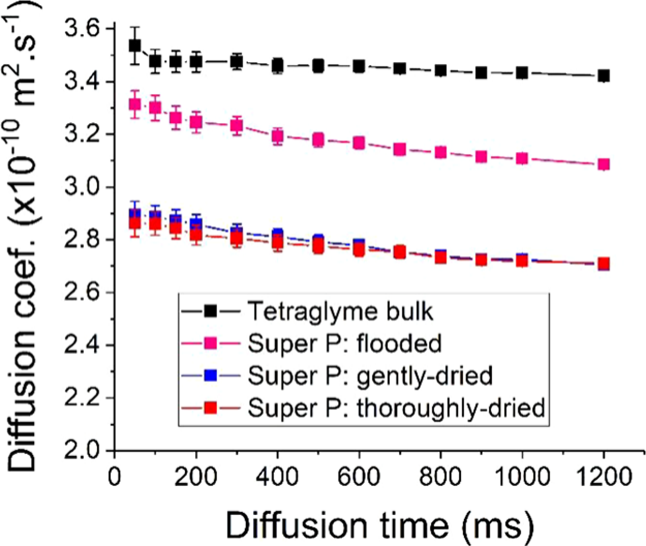
Diffusion coefficients for bulk tetraglyme and tetraglyme in three Super P electrodes, determined with PFG methods as a function of diffusion time, Δ.

The bulk diffusion coefficient in the pure tetraglyme solvent (*i.e.*, the value measured under nonconfined conditions) was first measured via ^1^H PFG NMR spectroscopy and determined to be 3.420 10^–10^ m^2^ s^–1^, with a 95% confidence range of ±0.005 10^–10^ m^2^ s^–1^ (*i.e.*, a 0.14% error bar; see Figure S2 for details). To measure the diffusion of tetraglyme confined in the porous structure of a Super P electrode, it is important that all the solvent molecules are inside the porous structure and that there are no molecules on the surface of the electrode since the latter will have larger effective diffusion coefficients than the confined ones and will affect the average measured diffusion coefficient. Two different drying approaches were tested. For the first one (the *gently dried* method), the soaked electrode is gently touched and wiped on both sides using a lab paper-towel. For the second drying approach (termed *thoroughly dried*), the *gently dried* electrode is put on a lab paper-towel and another piece of paper-towel is put on top of it. Then, a slight pressure was applied by hand to soak away any remaining liquid on the surface. ^1^H NMR spectra were acquired from samples prepared via the three approaches and used to estimate the relative solvent quantities (Figure S4) and show that the free electrolyte (sharp) signal was removed even after gently drying. Thoroughly drying removed further a 45% of the total solvent. Diffusion coefficient measurements on electrodes dried with the two techniques were then compared with results from two other samples: bulk tetraglyme; an electrode flooded with an excess of the tetraglyme solvent and is shown in Figure 6.

Unlike the neat tetraglyme, for samples containing the porous electrode, the effective diffusion coefficient decreases as the diffusion time increases, which is a signature of confined diffusion (Figure 3). The similar diffusion coefficients obtained for the *gently dried* and *thoroughly dried* samples provide confidence that both approaches are sufficient to remove the excess liquid from the surface of the electrodes, consistently with the ^1^H NMR spectra of the solvents (Figure S4). If that was not the case, the diffusion coefficients for the *gently dried* case would be higher than the *thoroughly dried* case as demonstrated by the results for the flooded sample. Even though a significant amount of additional electrolytes was removed (45%) in the *thoroughly dried* sample (vs the *gently dried* sample), this did not result in any visible effects from restricted diffusion due to empty pores with no solvent, as the diffusion coefficients measured at longer diffusion times for the *gently dried* and *thoroughly dried* were essentially identical. For subsequent experiments, the *gently dried* approach was used.

### Tortuosity Factor Evolution along Discharge

#### NMR

**Figure 7:**
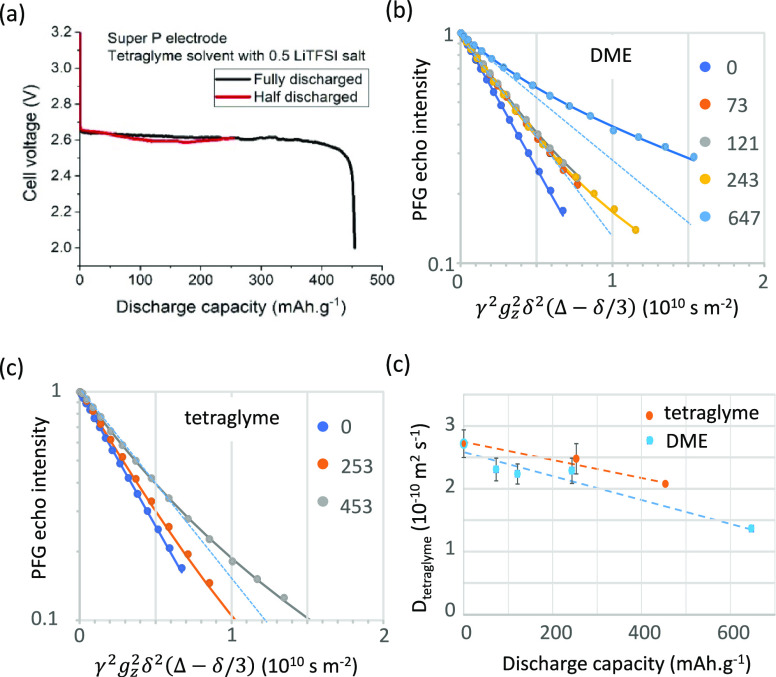
Tetraglyme diffusion in self-standing Super P electrodes as a probe for pore clogging at various states of discharge against lithium. (a) Constant 50 μA current discharge profiles of Super P with 0.5 M LiTFSI-tetraglyme as electrolyte. (b, c) Diffusion NMR of Super P electrodes discharged in (b) DME and (c) tetraglyme. ^1^H PFG NMR echo decays vs the composed experimental parameter γ^2^*g*_*z*_^2^δ^2^(Δ – δ/3) (eq 2) increasingly deviate from monoexponential behavior (dashed lines) at increasing depth of discharge. The data are well described in terms of a biexponential model (solid lines) with two discharge-constant diffusion coefficients and fractions, which vary with discharge. Phenomenologically, the two fractions represent the clog-free and clogged electrode parts. (d) Effective tetraglyme diffusion coefficients (a weighted average of the two diffusion coefficients) in self-standing Super P electrodes discharged with either DME or tetraglyme as electrolytes as a function of discharge. Dashed lines are a guide to the eye. The PFG NMR data shown here have been recorded for a diffusion time Δ = 1200 ms. For PFG echo decays at different Δ times, see Figure S5.

To study the tortuosity factor evolution along the depth of discharge, several self-standing Super-P electrodes were discharged at a discharge current of 50 μA. For the tetraglyme solvent, two electrodes are discharged: one cell is discharged to the end of capacity and the other is stopped halfway when a 253 mAh g^–1^ capacity is reached (Figure 7a). As there is some variability in the discharge capacity of Li-O_2_ batteries, the definition of “halfway” is only an approximation.^39,52^ For the DME solvent, four electrodes are discharged, and the discharge processes are stopped at 73, 121, 243, and 647 mAh g^–1^, the last one being the end of discharge.

All electrodes, whether discharged with tetraglyme or DME electrolyte, were dried and saturated with pure tetraglyme (without LiTFSI). Then, pore clogging was probed by measuring solvent diffusion at varied diffusion times in the range 20–1200 ms (Figure S5) using PFG NMR. Here, we focus on the data obtained with the longest diffusion time, Δ = 1200 ms where diffusion coefficients in pristine electrodes approach a convergence limit (Figure 6). The root-mean-square displacement <*r*^2^ >^1/2^ of bulk tetraglyme molecules indicates that electrolyte molecules travel approximately 50 μm in this time (<*r*^2^>^1/2^ = (6·*D*·*t*)^1/2^ = (6·*D*·Δ)^1/2^ = (6·3.42 10^–10^ m^2^ s^-1^·1.2 s)^1/2^ = 49.6 μm). This ensures that the diffusion coefficients do not measure local (*i.e.*, short range) motions but correspond to long-range diffusion.

Figure 7b,c and Figure S5 show PFG echo intensities versus the composite experimental variable *X* = γ^2^*g*_*z*_^2^δ^2^(Δ – δ/3) (γ, *g_z_*, δ, and Δ defined in eq 2). The observed decays for discharged electrodes deviate significantly from single-component behavior (eq 2) and increasingly so as the depth of discharge increases. The fast decay components reflect relatively unrestricted solvent diffusion, whereas the slow decay components represent the confined diffusion in clogged parts of the porous electrode. This heterogeneity also arises because of the geometry of the lithium-oxygen battery, oxygen entering from one side of the electrode and Li^+^ ions from the other. All PFG curves for tetraglyme diffusion in the electrodes at varied diffusion times and different stages of discharge in DME or tetraglyme are well described by a biexponential function *f*_A_exp(-*D*_A_*X*) + *f*_B_exp(-*D*_B_*X*). To improve the consistency of the analysis, all curves obtained for the same diffusion time, but at different states of discharge, are fit in a combined manner. The specific fit procedure treats the two diffusion coefficients as independent of the discharge stage and allows the components amplitudes to vary freely. While it is a simplification to divide the pores into two categories, clog-free and clogged with fast and slow diffusion, respectively, the model works well in a phenomenological way and can be used to explore distributions in diffusion coefficients.

**Figure 8:**
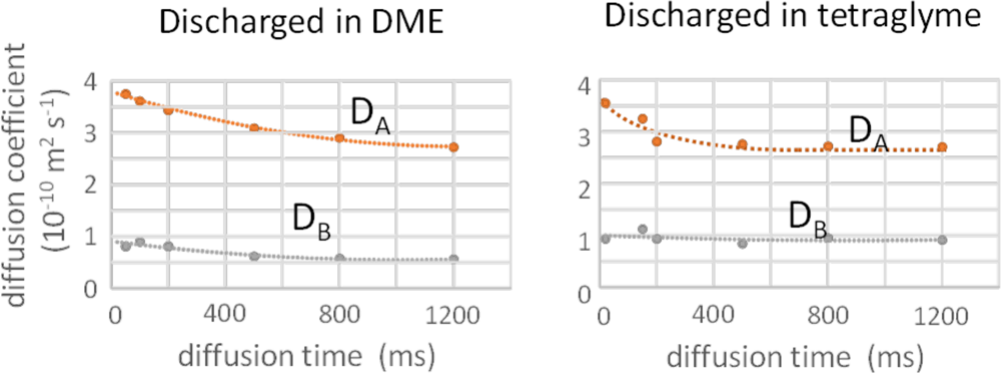
Diffusion coefficients pairs (*D*_A_, *D*_B_) extracted from bicomponent fits to PFG decays as a function of the diffusion time for an electrode discharged in DME (left) and tetraglyme (right). The biexponential model assumes that the values of the diffusion coefficients do not change with the discharge stage, but the relative fraction of each component does. Changes in pore clogging are reflected by the fraction of each component (Table 1).

Pairs of tetraglyme diffusion coefficients (*D*_A_, *D*_B_) extracted for electrodes discharged in DME and tetraglyme, as a function of diffusion time are plotted in Figure 8. The coefficient *D*_A_ of the mobile component decreases in both electrodes from close to the free tetraglyme diffusion value 3.5 10^–10^ m^2^ s^–1^ at short diffusion times toward 2.5 10^–10^ m^2^ s^–1^. This decrease likely indicates that even the mobile component in the largely un-clogged pores experiences diffusion restrictions at sufficiently long diffusion times. The *D*_A_ decrease also reflects the continuous solvent exchange between clog-free and clogged parts of the porous network within the timeframe of the measurement (0–1200 ms). The second coefficient *D*_B_ decays from ca. 1 10^–10^ to 0.51 10^–10^ m^2^ s^–1^ in the DME discharged electrode but is fairly constant around 1 10^–10^ m^2^ s^–1^ in the tetraglyme-discharged electrodes.

With the values of the diffusion coefficient pair (*D*_A_, *D*_B_) assumed independent of the discharge stage, the degree of pore clogging at varied discharge is reflected by the fraction *f*_B_ (representing the volume fraction of clogged pore) of the relatively immobile or restricted diffusion coefficient (Table 1). The fraction *f*_B_ in the bicomponent model generally increases with state of discharge, as expected, while for a given diffusion time *f*_B_, values are generally higher for the electrodes discharged in DME than in tetraglyme. Pore clogging thus appears to occur more frequently during discharge in DME. Interestingly, fraction *f*_B_ decreases with the diffusion time indicating that the diffusion appears to be more homogeneous at longer diffusion times as a larger pore volume is sampled. This may at least in part be ascribed to continuous solvent exchange between clog-free and clogged parts of the porous network, an effect that becomes more pronounced as the diffusion time increases.

**Table 1:** Fraction *f*_B_ of Immobile/Restricted Diffusion Component as a Function of Depth of Discharge and of the Diffusion Time, Tentatively Representing the Fraction of Clogged Pore Space

time	discharged in DME					discharged in tetraglyme		
	0 mA h g^–1^	73 mA h g^–1^	121 mA h g^–1^	243 mA h g^–1^	647 mA h g^–1^	0 mA h g^–1^	253 mA h g^–1^	454 mA h g^–1^
20 ms						0.16	0.36	0.32
50 ms	0.19	0.16	0.35	0.22	0.35			
100 ms	0.17	0.19	0.36	0.25	0.45			
150 ms						0.13	0.30	0.34
200 ms	0.19	0.18	0.32	0.24	0.53	0.00	0.20	0.24
500 ms	0.07	0.17	0.26	0.21	0.54	0.00	0.13	0.26
800 ms	0.04	0.14	0.24	0.21	0.60	0.00	0.15	0.33
1200 ms	0.00	0.19	0.22	0.20	0.65	0.00	0.13	0.36

The tortuosity model treats the porous network as homogeneous, whereas the bicomponent model artificially divides the pore network into two categories, clog-free and clogged pores. In reality, of course, various degrees of pore clogging may occur in different pores, and dynamic solvent exchange between clog-free and clogged pore areas may also reduce the contrast between these regions.

A possible link between the tortuosity model and bicomponent model can be established by deriving the weighted average diffusion coefficient *D*_av_ = *f*_A_*D*_A_ + *f*_B_*D*_B_ from the bicomponent fit (Table S1). For the biexponential model used in our analysis, this corresponds to the initial slope of the decay. This represents an alternative way to characterize the PFG curves with a single effective diffusion parameter. The average diffusion coefficient *D*_A_ is always lower than the free tetraglyme diffusion coefficient and generally decreases with discharge and the overall diffusion time, as expected. The weighted average diffusion coefficient from the biexponential model for a diffusion time of 1200 ms is shown on Figure 7d and can be compared with the monocomponent fitting shown on Figure S5. The diffusion coefficients have the same trends in both cases. However, the errors in the bicomponent fitting are significantly lower (the single-component fit showing errors as large as 60%, see the Supporting Information for the error analysis, and bicomponent fit leading to errors below 20%), supporting our analysis of the diffusion in terms of more than one diffusion coefficient. Our simple analysis considers two types of diffusion in the electrode: one in more restricted (clogged pores) and the other in less restricted (clog-free) porous domains. In reality, the electrode will have a distribution of diffusion domains with varying degrees of restriction (clogging).

The overall trend observed for both solvents is that the effective diffusion coefficient decreases as the cell is discharged further and the transport through the electrode becomes more tortuous. This is consistent with the growth of discharge products leading to a reduction of porosity via pore shrinkage and clogging of some pores. Higher effective diffusion coefficients are measured for the electrodes discharged in the tetraglyme-based electrolyte compared to the electrodes discharged in the DME-based electrolyte. This is ascribed to the larger particles formed and more pore clogging in DME: DME has a higher donor number than tetraglyme, and thus, we expect the corresponding cells to show larger Li_2_O_2_ particles than the tetraglyme ones.^22,23^

**Figure 9:**
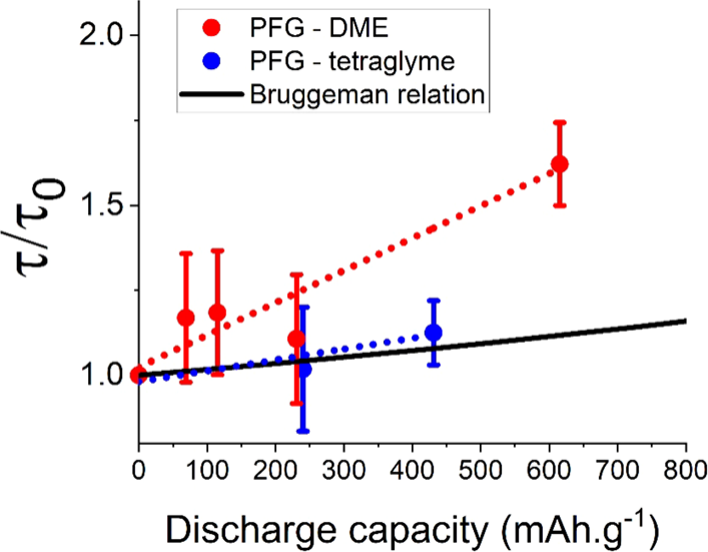
Tortuosity factors in pristine, partially, and fully discharged self-standing Super P electrodes, extracted from PFG-NMR measurements using weighted average diffusion coefficients from the biexponential model. The solvent used for discharge is indicated in the legend. The Bruggeman relation is applied by assuming that all discharge products are Li_2_O_2_. Dashed lines for experimental data are linear fit to the data points weighted with the error bars. An explanation of the error analysis is given in the Supporting Information.

The evolution of the tortuosity factor can be calculated from the effective diffusion coefficients using eq 3. The porosity of the pristine electrode is 0.81, as calculated from the measured electrode dimensions and mass (see Table S2 in the Supporting Information). For this calculation, we assume that the only discharge product is Li_2_O_2_, which allows the volume of Li_2_O_2_ to be calculated from the discharge capacity and the change of porous volume, and porosity, to be estimated. The evolution of the tortuosity factor was then estimated via Bruggeman relation using eq 10
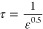
10and compared with that obtained from the PFG data in Figure 9.

The tortuosity factor values extracted from PFG-NMR measurements for DME change more drastically than suggested by the Bruggeman relation, particularly for DME.^53^ The tortuosity factor evolution depends on the porosities of the pristine and discharged electrodes, which are only estimated here. Thus, we have also plotted  vs depth of discharge and is shown in Figure S6 since this does not require any assumptions concerning the change in porosity: it is simply the ratio of *D*_eff_/*D*_0_ (see eq 3). The plot shows the same trends as Figure 9, supporting our analysis of tortuosity vs depth of discharge.

#### Tomography

To measure the change of tortuosity factor with a second technique, tomographic imaging was conducted on two samples: partially (206 mAh g^–1^) and fully (362 mAh g^–1^) discharged Super P electrodes with 0.5 M LiTFSI in tetraglyme electrolytes. Tortuosity factor values of 4.95 and 5.4 are calculated from the tomographic images for partially and fully discharged cells, respectively. To compare them with the evolution of tortuosity factor values measured by PFG-NMR experiments, we compare the rate of change of the tortuosity factor, *i.e.*, the slopes of the discharge capacity versus  plots shown in Figure 9 (Table 2). Although the errors associated with the PFG measurements are large, which is largely ascribed to differences between cells, each *ex situ* PFG measurement was made on a different cell, and errors in the PFG measurements (as discussed above) associated with describing the PFG data with a single diffusion coefficient for the fully discharged sample, a significant difference is seen between the values calculated for the tetraglyme and DME-discharged electrodes. The tortuosity factor value evolution calculated from tomographic images for electrodes discharged with tetraglyme electrolyte is close to the value measured by PFG-NMR experiments for the same electrodes and very different from the electrodes discharged in DME-based electrolyte and the Bruggeman estimation.

**Table 2:** Rate of Change of the Tortuosity Factor along Discharge, Calculated with Three Different Techniques

sample and method	rate of tortuosity factor change along discharge × 10^–4^ (g m^–1^A^–1^ h^–1^)
Bruggeman relation	2.00 ± 0.03
PFG-NMR tetraglyme	7.5 ± 2.1
PFG-NMR DME	16.6 ± 2.5
TXM tetraglyme	5.9

**Figure 10:**
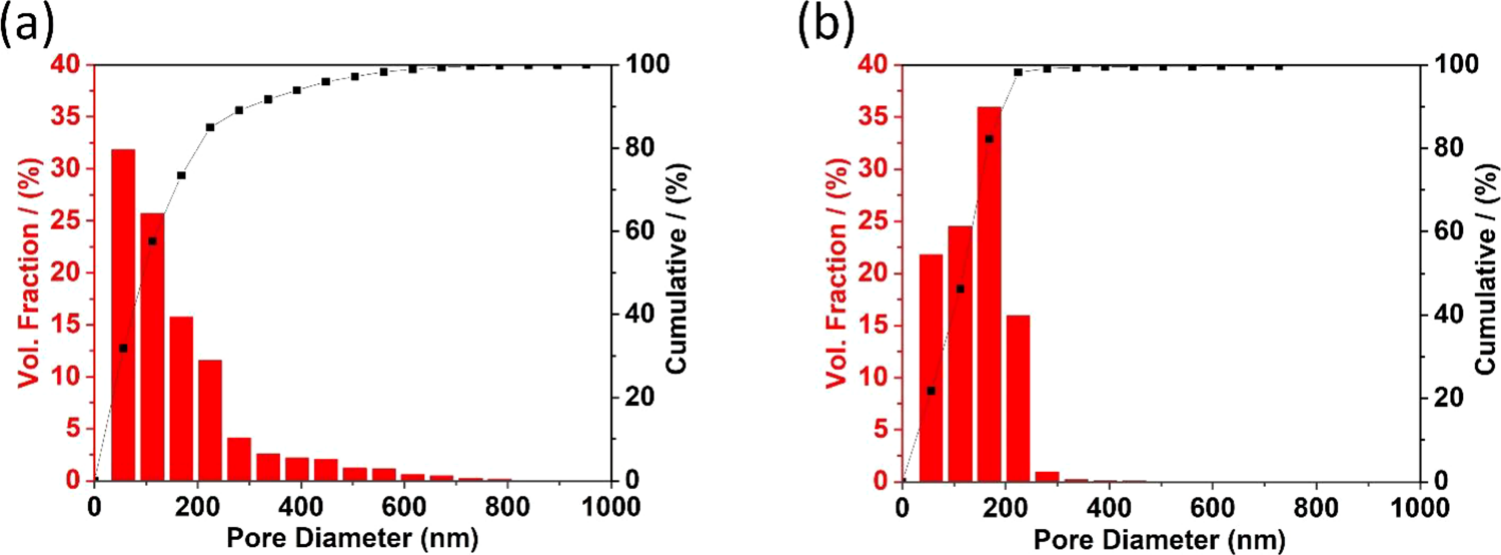
Pore size distributions obtained from tomographic images of (a) partially 206 mAh g^–1^ and (b) fully 362 mAh g^–1^ discharged Super P electrodes with 0.5 M LiTFSI, tetraglyme electrolytes at 50 μA discharge current.

The analysis of the tomographic images also allows us to extract pore size distributions, which show an overall increase in the fraction of smaller pores, suggesting a shrinkage of the pores (Figure 10). However, clogging of micropores with pore sizes below 54 nm is hard to evaluate solely by tomography measurements due to imaging resolution, and any analysis should be coupled with other techniques.

### PNM Modeling

The PNM approach^39^ was then used to explore the hypothesis that the formation of larger discharge particles leads to more pore clogging. In the PNM approach, the escape factor parameter, χ, can be varied to adjust the relative contributions of the film growth mechanism versus the particle growth mechanism. A larger value of χ corresponds to a more predominant particle growth mechanism and thus the formation of larger particles of Li_2_O_2_.

**Figure 11:**
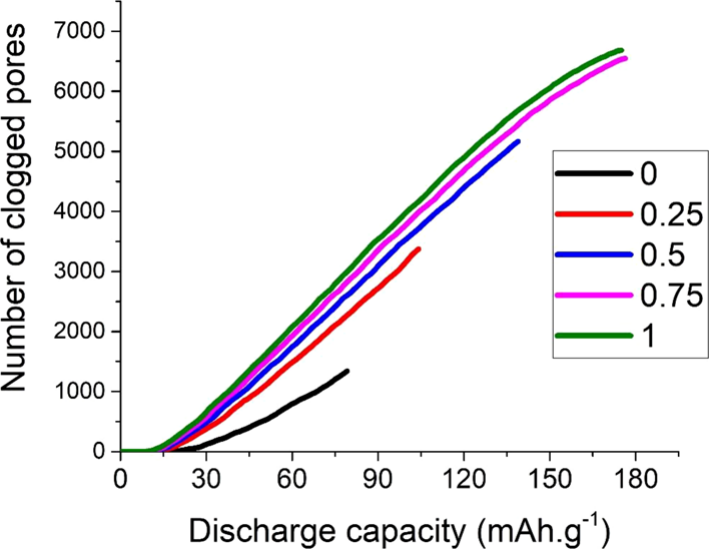
Number of clogged pores for the Super P electrode structure calculated using PNM modelling of the pristine Super P electrode structure obtained from tomographic imaging. The discharge current density is 100 μA g^–1^. Plots are shown for five escape factors (χ =0, 0.25, 0.5, 0.75, and 1).

Figure 11 shows the evolution of the number of clogged pores along discharge for five different escape factors, the number of clogged pores being higher for larger escape factors. This calculation thus provides a plausible interpretation as to why the effective diffusion coefficients for electrodes discharged in DME-based electrolytes decreases faster along discharge. It is worth noting that there is a large difference between the curves for χ = 0 and χ = 0.25 but the changes are more limited for higher escape factors. This would indicate that even without being predominant, the onset of particle growth is enough to significantly enhance the pore clogging phenomenon. Indeed, as shown in Figure S7 (the gradient in discharge products is shown in Figure S8), already for χ = 0.25, a large number of particles of 25 μm or larger are produced; these particles are sufficiently large to clog the majority of the pristine Super P carbon pores with a size between 25 and 50 μm. As a consequence, the larger particles produced at higher χ values result in less dramatic changes.

As was shown in a previous work,^40^ PNM calculations on three-dimensional electrode structures also yield larger tortuosity factor changes on discharge compared to the Bruggeman relation. The larger increase in the tortuosity factor for PNM calculations is due to the incorporation of pore clogging effects in the simulations of 3D electrode structures. Similarly here, pore clogging could be the reason why a more dramatic tortuosity factor change is observed in PFG-NMR measurements compared to the Bruggeman relation.

### kMC Modeling

Another factor that can affect the effective diffusion coefficient of a molecule in a porous media is the frequent collisions of particles with the pore wall. This phenomenon, which is called Knudsen diffusion, is particularly important when the pore is very small. This effect is naturally present in the effective diffusion coefficient measurements done with PFG-NMR experiments. Yet in most cases, the tortuosity factor is considered only as a function of porosity. Here, to quantify the effect of pore size on diffusion, a kMC model is used.

**Figure 12:**
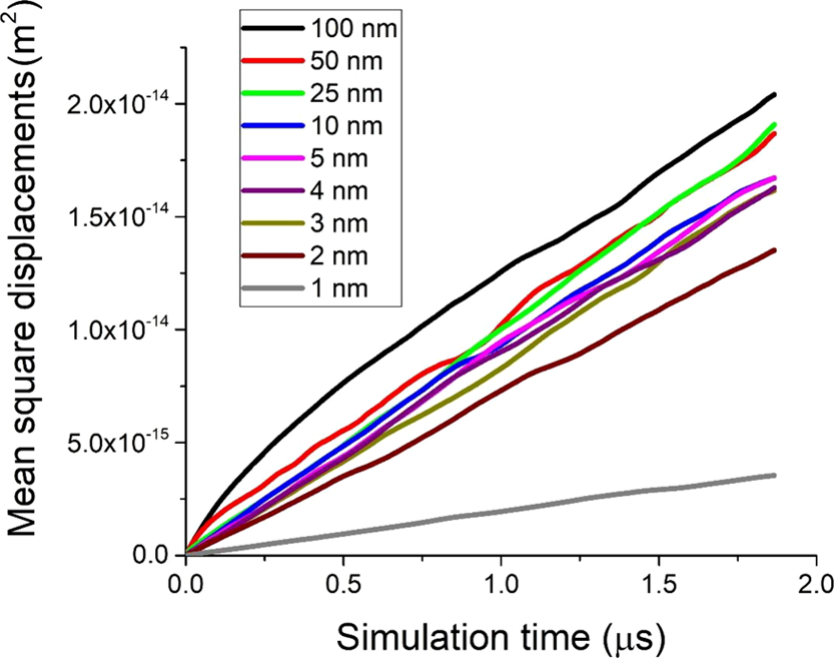
MSD plots obtained from kMC calculations for several pore sizes.

To assess the importance of this effect, the bulk diffusion coefficient was given as an input and set to 5·10^–9^ m^2^ s^-1^, which is within the typical range of oxygen diffusion coefficients reported for Li-O_2_ battery electrolytes.^22^ Oxygen diffusion is studied first before changing the diffusing molecule to tetraglyme molecules. Nine different pore sizes were tested, corresponding to cylindrical pores with diameters of 1, 2, 3, 4, 5, 10, 25, 50, and 100 nm, which are within the range of Super P pore sizes.^8^ The obtained MSD curves are shown in Figure 12 (and Figure S9 for *x*, *y*, and *z* contributions to the overall diffusion). The MSD values increase nonlinearly with time for short times but become linear after 1 μs. This is particularly visible for the 100 and 50 nm pore sizes.

**Figure 13:**
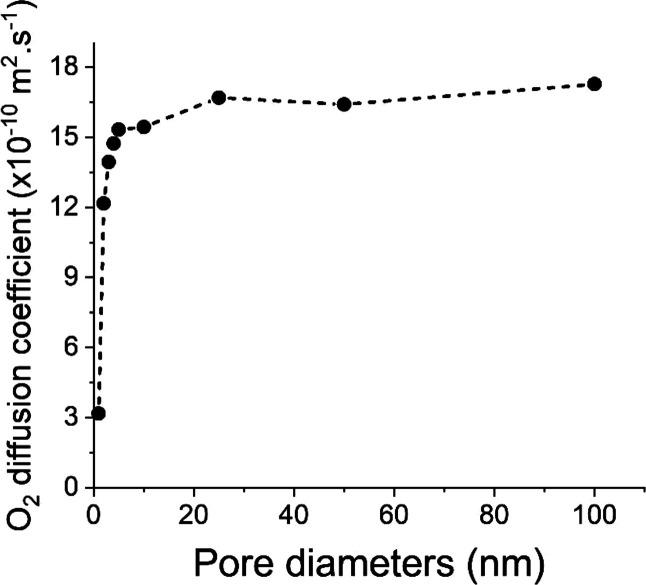
O_2_ diffusion coefficients calculated using the kMC model for various pore diameters.

The diffusion coefficient as a function of the pore diameter calculated from kMC trajectories is shown in Figure 13. While the effective diffusion coefficient does not vary much for pores with diameters larger than 25 nm, a pore size effect is seen below 25 nm. The effective diffusion coefficient decreases sharply for pores with diameters below 4–5 nm.

The sensitivity of these trends on the input diffusion coefficient value and voxel size was analyzed. As the molecule tracked in the PFG-NMR experiments is tetraglyme, two similar kMC calculations were done with tetraglyme as the diffusing molecule instead of O_2_. For the first of these two additional kMC calculations, the input value for the bulk diffusion coefficient was simply changed from the value of O_2_ to the tetraglyme bulk diffusion coefficient (measured by PFG-NMR and equal to 3.42 10^–10^ m^2^ s^–1^). In the second calculation the voxel size was changed from 0.5 to 0.75 nm (to represent the longer chain of tetraglyme molecules). The diffusion coefficient value does not change the pore size effect on the effective diffusion coefficient values, while the voxel size seems to affect the effective diffusion coefficient values only slightly (Figure S10).

These three kMC calculations confirm that the pore size effect on the effective diffusion coefficient is not noticeable for pores larger than 5 nm and only significant below that size. These trends are consistent with PFG-NMR measurements conducted by Forse *et al.*(35) for self-diffusion coefficients of ions in activated carbons for supercapacitors. They measured effective diffusion coefficients about two orders of magnitude lower for ions in nanopores compared to the bulk values. These kMC calculations and prior reports on the pore size effect on the effective diffusion coefficient confirm our assumption that the change in the measured effective diffusion coefficient values are mainly influenced by pore clogging in the electrode mesostructure.

## Summary and Conclusions

In this study, several Super P electrodes were discharged to various depths of discharge and effective diffusion coefficients of tetraglyme molecules in the porous cathodes were measured using PFG-NMR experiments. This allowed for the extraction of the tortuosity factor evolution along discharge for Li-O_2_ batteries. It was shown that the tortuosity factor increases with the depth of discharge. The effect of the solvent on the tortuosity factor evolution of Super P electrodes was also studied. When a DME-based electrolyte is used, the tortuosity factor increases more dramatically than when tetraglyme is used as the electrolyte solvent. The tortuosity factor evolution along discharge is confirmed with tomographic imaging for cells discharged with tetraglyme electrolytes. This difference between solvents is attributed to the difference in the size of discharge products in the two electrolytes.

PNM and kMC calculations were used to investigate the relative importance of pore clogging and pore shrinking on the effective diffusion coefficients. PNM calculations showed that electrolytes with larger escape factor (higher donor number) lead to more pore clogging. This provides a plausible reason for why the tortuosity factor of the electrodes discharged in DME based electrolyte increases more than seen in tetraglyme. The kMC calculations show that there is indeed a pore size effect on diffusion, but it manifests itself only for very small pores with diameters below 5 nm. In Super P electrodes, less than 0.1% of the porous volume correspond to pores with radiuses below 90 nm, which makes this effect negligible in these electrodes.^12^ In the future, these results could be integrated in the PNM model to improve its accuracy by including the pore size effect on diffusion; the PNM would capture in a better way the influence of the pore shrinkage along discharge.

These experimental and modeling results can be used to suggest ways of optimizing battery performance. For instance, using an electrolyte that promotes formation of large discharge products can provide high discharge capacities but may not perform well at high discharge rates as the pore clogging will hinder ionic transport. On the contrary, electrolytes in which small particles are favored reduce pore clogging and favor faster transport. Porous carbon materials and electrolytes thus have to be optimized in parallel to have particle sizes large enough for a high capacity but without clogging the electrode pores. The measured rate of change of tortuosity could in future work be used to optimize cell performance. For instance, there are several factors, such as high discharge current, that could lead to smaller Li_2_O_2_ particle sizes. The ability to measure the electrode tortuosity after the cells have been discharged allows one to optimize the discharge rate to maximize capacity while still maintaining a low tortuosity. Furthermore, it is potentially possible to determine whether shut-down of the cell is more likely due to a decrease in the overall tortuosity or to pore blockage on the air side of the electrode.

## Supporting Information Available

More details on PFG-NMR measurements, error analyses, calculating diffusion coefficients from kMC, and sensitivity analysis for kMC data are provided (PDF)
