# Supplementary material for: Probing and Interpreting the Porosity and Tortuosity Evolution of Li-O2 Cathodes on Discharge through a Combined Experimental and Theoretical Approach
Source: J Phys Chem C Nanomater Interfaces. 2021 Feb 25;125(9):4955–67. doi: 10.1021/acs.jpcc.0c10417 (PMC7976606; doi:10.1021/acs.jpcc.0c10417)
Supplement: Supplementary file 1 — jp0c10417_si_001.pdf [file jp0c10417_si_001.pdf]

Supporting information for

Probing and Interpreting the Porosity and Tortuosity Evolution of Li-O_2 Cathodes on Discharge Through a Combined Experimental and Theoretical Approach

*Amangeldi Torayev,[1,2,3] Simon Engelke,[2,4] Zeliang Su,[1,5] Lauren E. Marbella,[2,6] Vincent De Andrade,[7] Arnaud Demortiere,[1,3,5] Pieter C. M. M. Magusin,[2,3] Céline Merlet,[3,5,8] Alejandro A. Franco,[1,3,5,9] Clare P. Grey [*2,3]*

[1] *Laboratoire de Réactivité et Chimie des Solides (LRCS), UMR CNRS 7314, Université de Picardie Jules Verne, Hub de l'Energie, 15 Rue Baudelocque, 80039 Amiens, France*

[2] *Department of Chemistry, University of Cambridge, Lensfield Road, Cambridge CB2 1EW, United Kingdom*

[3] *ALISTORE-European Research Institute, FR CNRS 3104, Hub de l'Energie, 15 Rue Baudelocque, 80039 Amiens, France*

[4] *Cambridge Graphene Centre, University of Cambridge, 9 JJ Thomson Avenue, Cambridge, CB3 0FA, UK*

[5] *Réseau sur le Stockage Electrochimique de l'Energie (RS2E), FR CNRS 3459, Hub de l'Energie, 15 Rue Baudelocque, 80039 Amiens, France*

[6] *Department of Chemical Engineering, Columbia University, 500 W 120th St, New York, NY 10027, USA*

[7] *X-Ray Science Division, Advanced Photon Source, Argonne National Laboratory, Lemont, USA*

[8] *CIRIMAT, Université de Toulouse, CNRS, Bât. CIRIMAT, 118, route de Narbonne 31062 Toulouse cedex 9, France*

[9] *Institut Universitaire de France, 103 Boulevard Saint-Michel, 75005 Paris, France*

*cpg27@cam.ac.uk

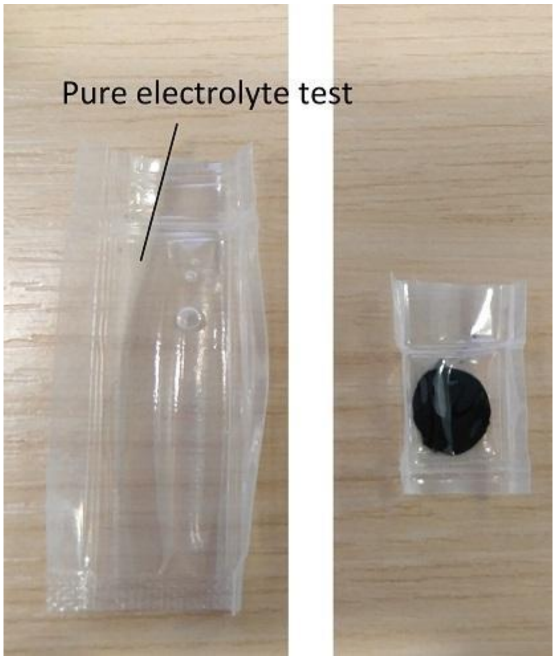

Figure S1. *Sample holders for PFG-NMR experiments.*

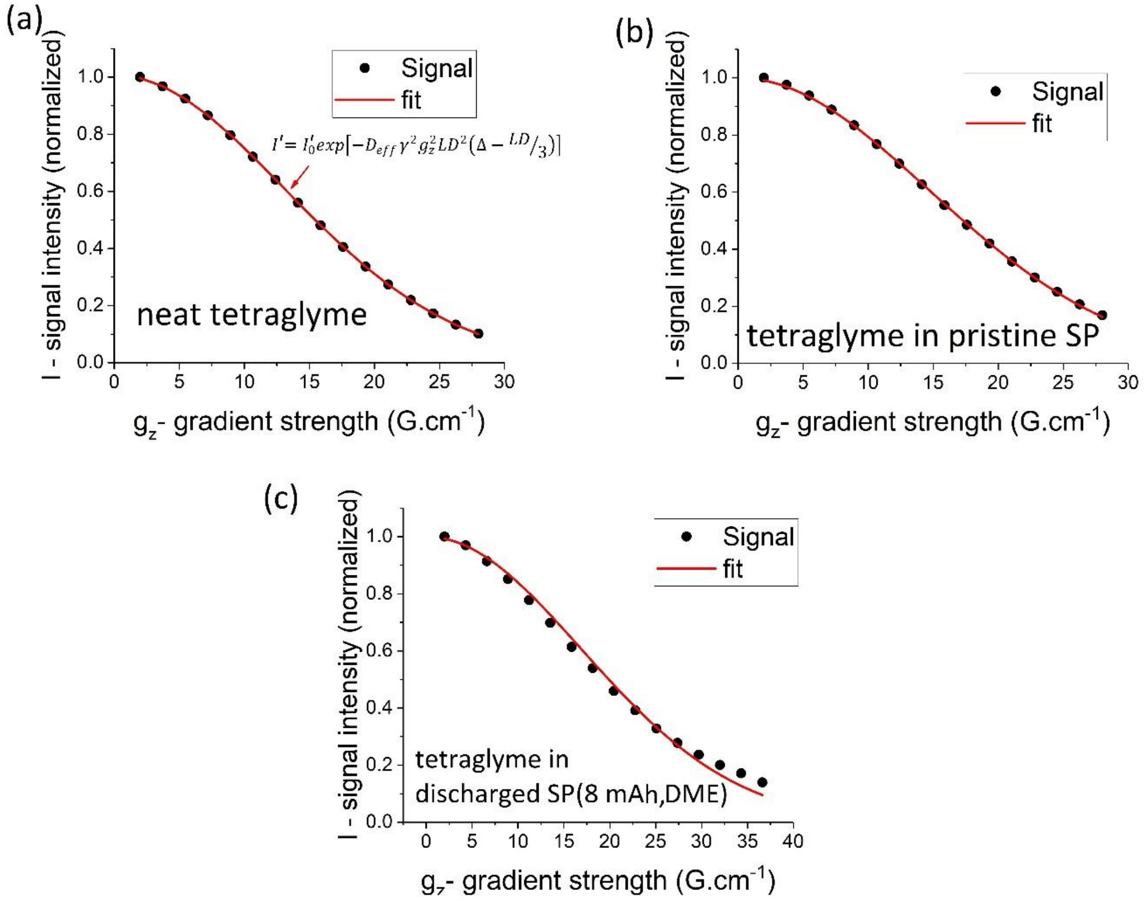

Figure S2. *PFG-NMR signal intensities recorded for various gradient strengths for (a) neat tetraglyme (b) tetraglyme in pristine Super P electrode (c) tetraglyme in fully discharged (with 0.5 M LiTFSI in DME) Super P.* Δ = 1200 ms; δ = *1 ms. The signal follows the Stejskal-Tanner equation which describes its intensity as a function of the gradient strength and the diffusion coefficient. The diffusion coefficient is thus extracted by fitting to the acquired signal intensities.*

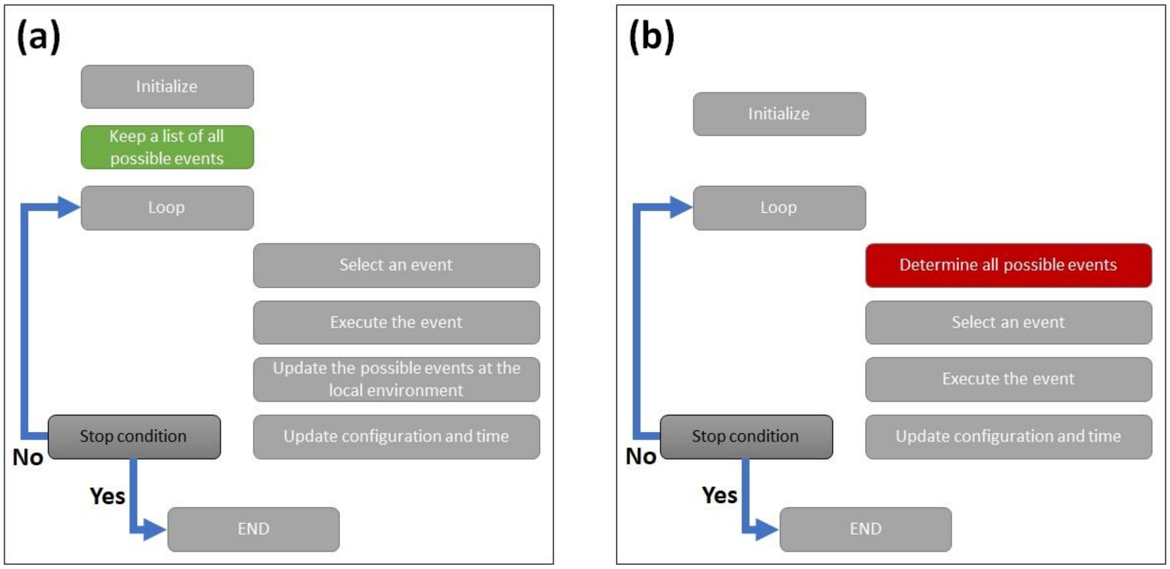

Figure S3. *kMC model flow chart used in (a) this work and (b) literature by Blanquer et al.*[1] *and Reuter*[2]*.*

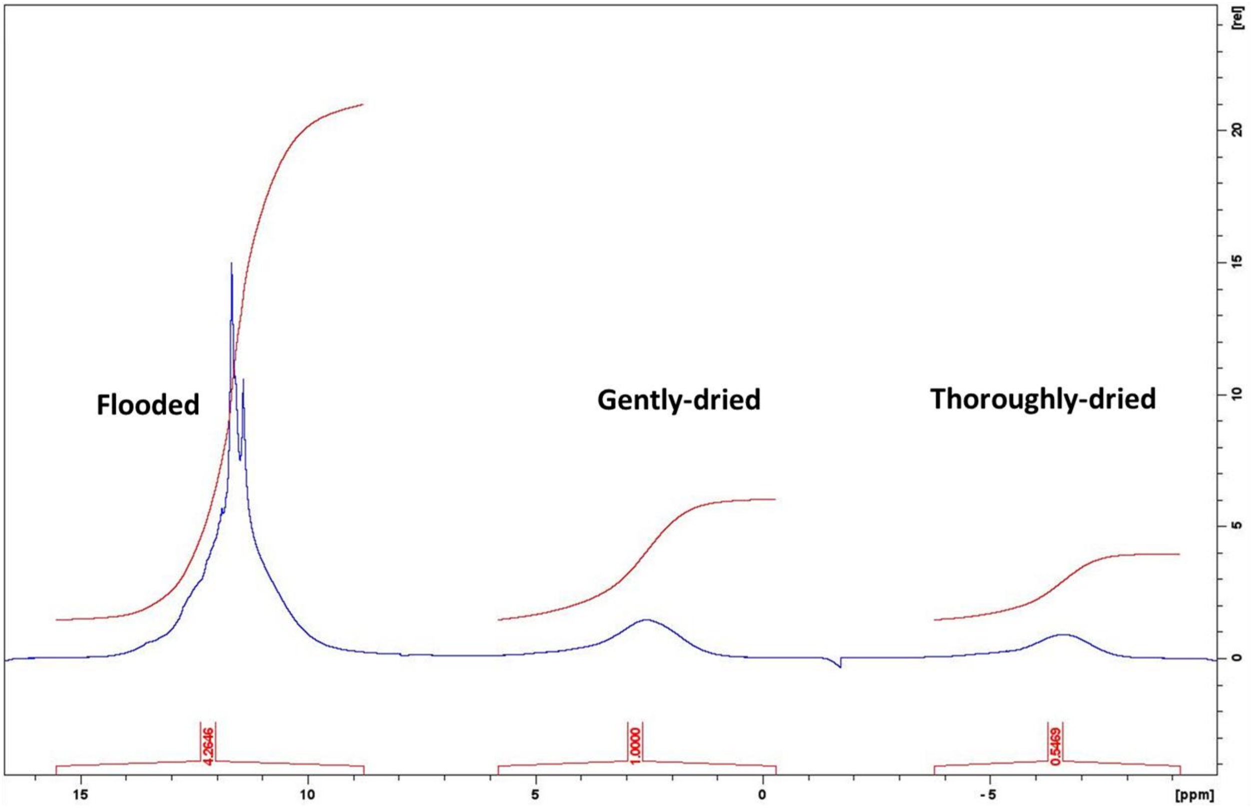

Figure S4. *Relative solvent quantities for three types of dried electrodes (flooded: 4.26, gently-dried: 1, thoroughly-dried: 0.55).*

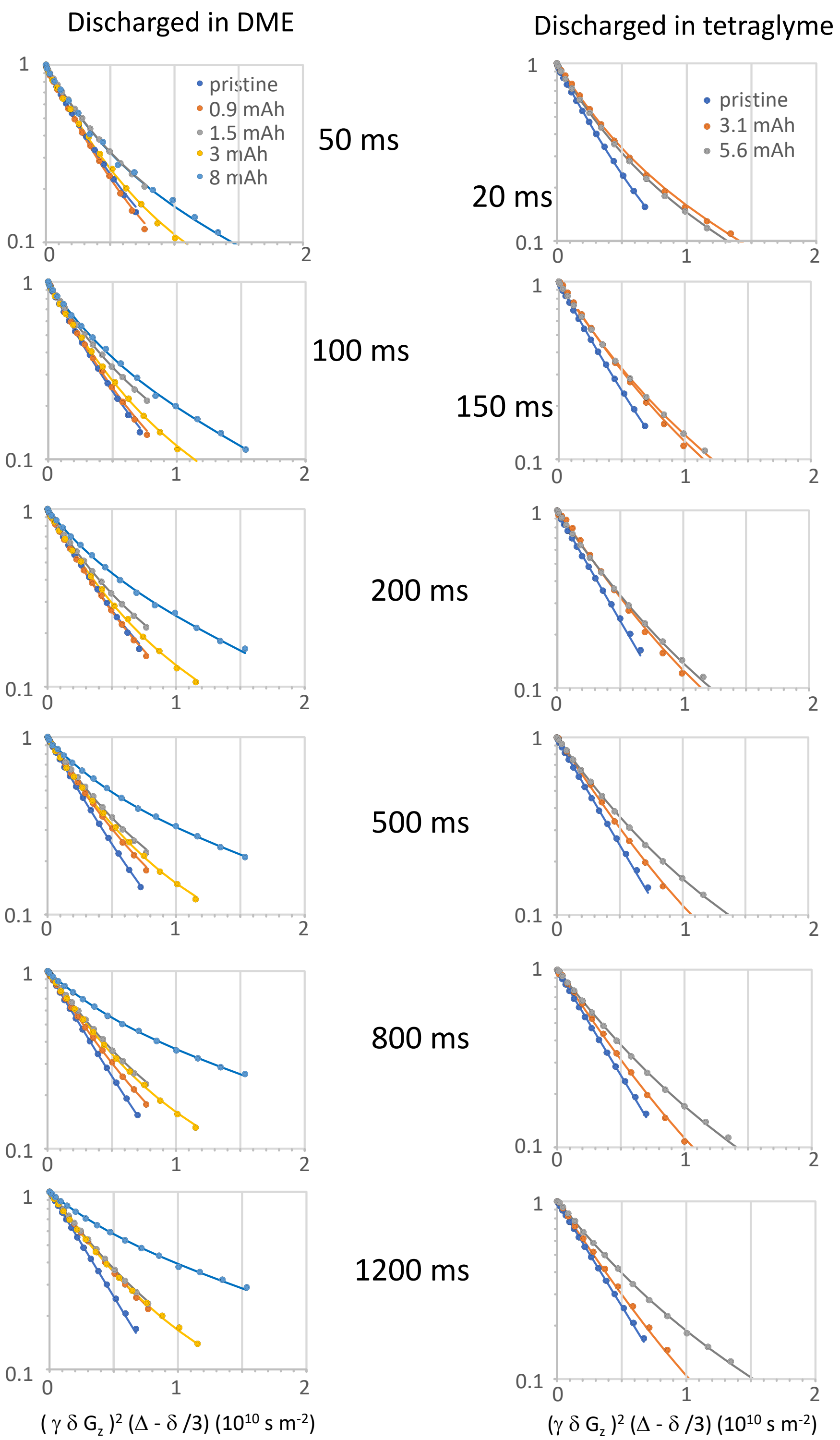

Figure S5. *PFG echo intensities plotted versus the composed experimental parameter* $\gamma^2 g_z^2 \delta^2(\Delta - \delta/3)$ *(Eq. 2)*

		Discharged in DME					Discharged in tetraglyme		
		0 mAh.g^{-1}	73 mAh.g^{-1}	121 mAh.g^{-1}	243 mAh.g^{-1}	647 mAh.g^{-1}	0 mAh.g^{-1}	253 mAh.g^{-1}	454 mAh.g^{-1}
	20 ms						3.14	2.62	2.71
	50 ms	3.21	3.28	2.73	3.09	2.73			
	100 ms	3.16	3.10	2.65	2.95	2.39			
	150 ms						2.98	2.61	2.52
	200 ms	2.94	2.96	2.60	2.79	2.04	2.81	2.44	2.36
	500 ms	2.91	2.66	2.43	2.56	1.76	2.76	2.50	2.26
	800 ms	2.80	2.58	2.34	2.42	1.49	2.72	2.45	2.14
	1200ms	2.72	2.31	2.24	2.29	1.32	2.71	2.48	2.07

Table S1. *Weighted average diffusion coefficients $D_{av} = f_A D_A + f_B D_B$ derived from the bi-exponential model fitting in units of 10^{-10} $m^2.s^{-1}$.*

Error analysis:

The error calculation for τ_i/τ_0 is calculated as follows. The main relation is

$$D_i = \frac{\varepsilon_i}{\tau_i} D_{bulk}$$

or

$$\tau_0 = \varepsilon_0 \frac{D_{bulk}}{D_0} \quad \text{and} \quad \tau_i = \varepsilon_i \frac{D_{bulk}}{D_i}$$

The relation for τ_i/τ_0 is

$$\frac{\tau_i}{\tau_0} = \frac{\varepsilon_i \frac{D_{bulk}}{D_i}}{\varepsilon_0 \frac{D_{bulk}}{D_0}} = \frac{\varepsilon_i \cdot D_{bulk} \cdot D_0}{\varepsilon_0 \cdot D_{bulk} \cdot D_i} = \frac{\varepsilon_i}{\varepsilon_0} \cdot \frac{D_0}{D_i}$$

Table S2. Data related to the calculation of the electrode porosity

**thickness**	300	µm
**diameter**	11	mm
**70% Super P**	2.26	g cm^3
**30% PTFE**	2.2	g cm^3
**electrode mass**	13	mg cm^2
**For a 1 cm^2 electrode surface**		
**electrode volume**	30 x 10^{-3}	cm^3
**mass**	13 x 10^{-3}	g
**Super P weight**	9.1 x 10^{-3}	g
**PTFE weight**	3.9 x 10^{-3}	g
**SP volume**	4.03 x 10^{-3}	cm^3
**PTFE volume**	1.77 x 10^{-3}	cm^3
**solid volume**	5.80 x 10^{-3}	cm^3
**porosity**	0.81	

$$Porosity = \frac{Electrodevolume - PTFEvolume - SPvolume}{Electrodevolume}$$

The errors for the diffusion coefficients are obtained from the fitting of the Stejskal-Tanner equation to the PFG-NMR signal as shown in Figure S2. The error for D_0 is 0.49% and the error for D_i differs for each cell i ranging from 0.49% for pristine Super P electrode to 32.17% for fully discharged Super P electrode with DME based electrolyte. As all the discharge capacity is coming from the formation of Li_2O_2 discharge products, the error in porosity is assumed to be zero. The error for τ_i/τ_0 is thus the sum of the maximum errors on D_0 and D_i.

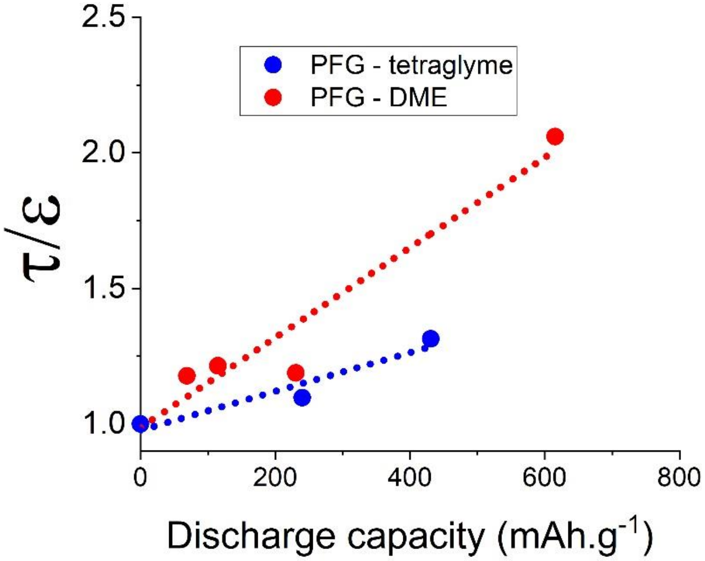

Figure S6. $\frac{\tau}{\varepsilon}$ *evolution as a function of depth of discharge.*

Calculating the tortuosity evolution along the depth of discharge using the measured diffusion coefficients requires known porosity values (ε) at these discharge points to use the equation below (which corresponds to equation 3 in the main paper):

$$D_{eff} = \frac{\varepsilon}{\tau} D_{bulk} \quad \text{or} \quad \tau = \varepsilon \frac{D_{bulk}}{D_{eff}}$$

In Figure 8 in the main text, the initial porosity is calculated using Table S2 and the porosity change along the depth of discharge is calculated from the capacity by assuming all the discharge product is Li_2O_2. In order to remove any potential errors that arise from the estimate of the porosity, ε, we can simply compare τ/ε as a function of state of discharge. Thus the data in Figure 8 in the main text has been replotted in Figure S6 with τ/ε on the y-axis, which simply reflects the ratio of the bulk diffusion and effective diffusion coefficients at each depth of discharge as measured by PFG-NMR. The same trends are observed in Figure S6 and 8, confirming that the trends observed for the tortuosity change are not affected by the porosity assumption.

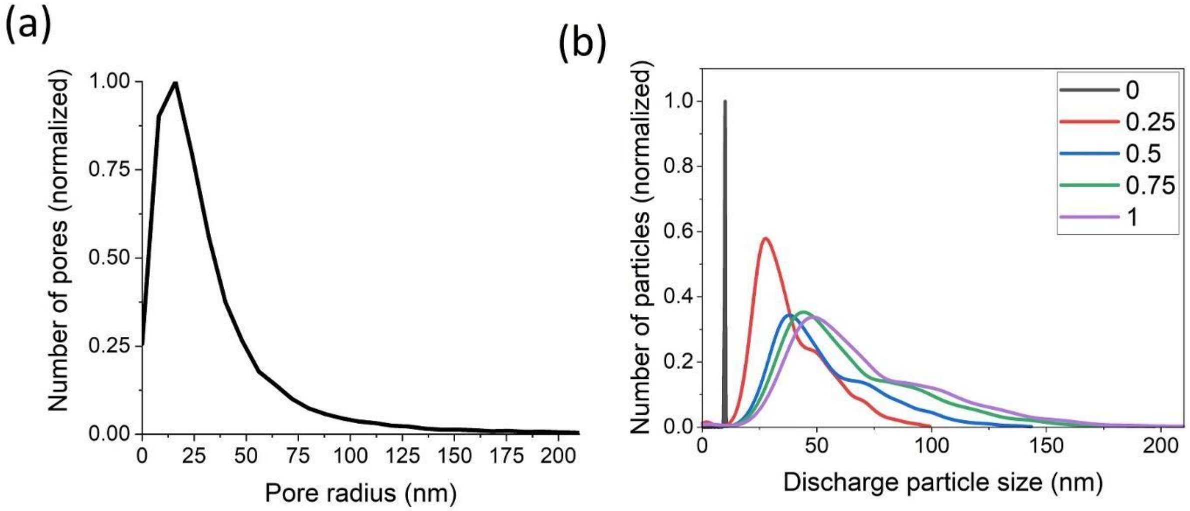

Figure S7. *(a) Pore size distribution for the Super P carbon used for the PNM model; (b) discharge product size distribution at the end of discharge for 5 different escape factors.*

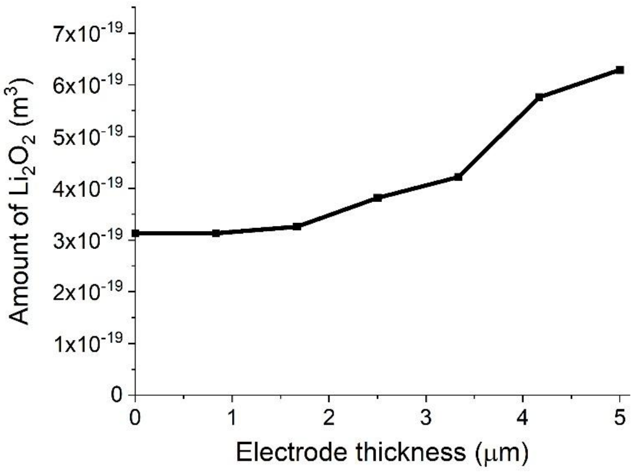

Figure S8. *Discharge product gradient at the end of discharge for escape factor 0, PNM model*

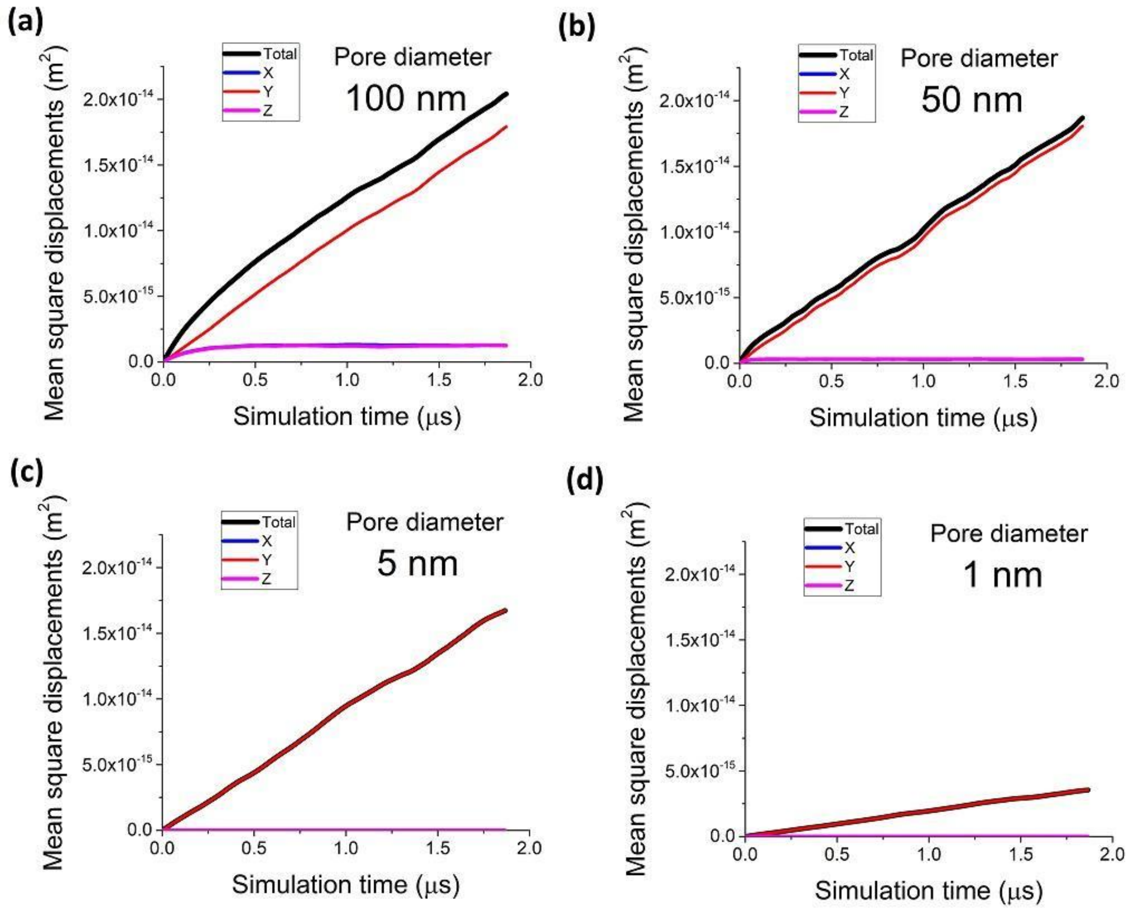

Figure S9. *MSD curves calculated from kMC trajectories in x, y and z directions for pores with (a) 100 nm, (b) 50 nm, (c) 5 nm and (d) 1 nm diameters.*

Figure S9 shows MSD plots calculated from kMC trajectories. The MSD curves for the x and z directions reach a plateau as expected for confined diffusion.[3] On the other hand, the MSD along the y direction reaches a linear regime without plateau since there are no restrictions along this direction. The diffusion coefficients are obtained by fitting the MSD values in the y direction. Monte Carlo simulations are stochastic in nature and several simulations are needed to ensure a sufficient statistical accuracy. For each structure, the kMC calculations are repeated three times; and we report the average of those three repetitions.

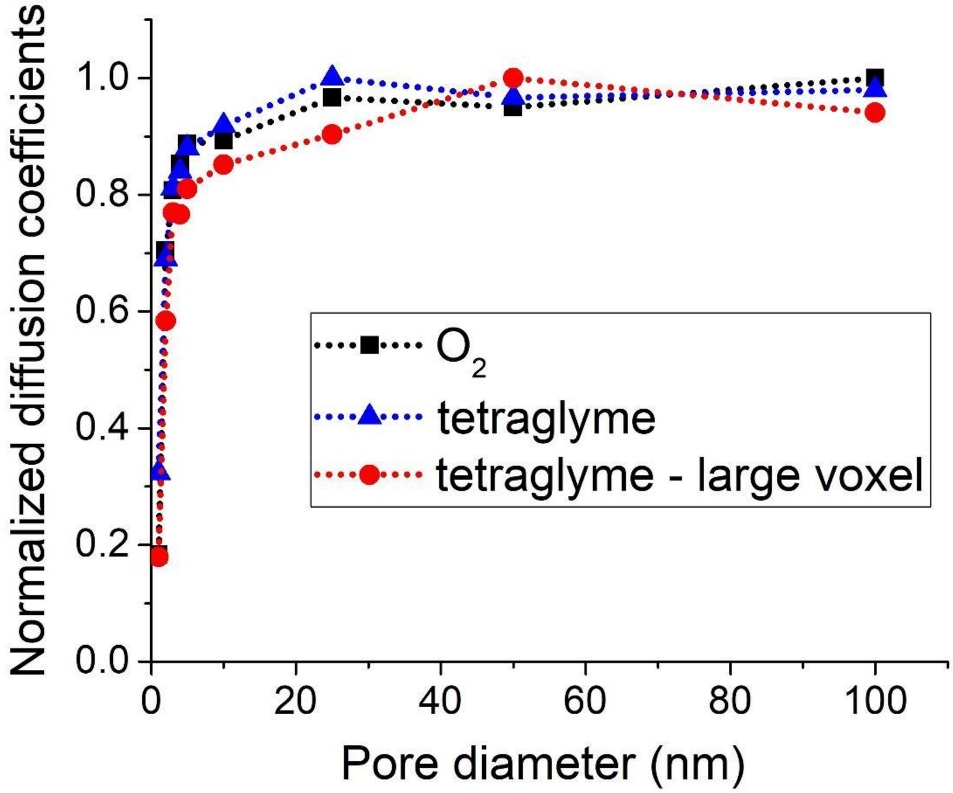

Figure S10. *O_2 and tetraglyme effective diffusion coefficients calculated using the kMC model for various pore diameters. For the tetraglyme case, two voxel sizes are tested, one with 0.5 nm and another with 0.75 nm (labeled as "tetraglyme - large voxel").*

The marginal differences between diffusion coefficients obtained with different voxel sizes are largely artifacts of the model which arise because of the mapping of a cylinder with square voxels. When the voxel size gets smaller, it maps more area within the cross-section of the cylinder. Thus, when the molecule size gets larger (which corresponds to larger voxels), the molecule has marginally less area to diffuse within.

REFERENCES

(1) Blanquer, G.; Yin, Y.; Quiroga, M. A.; Franco, A. A. Modeling Investigation of the Local Electrochemistry in Lithium-O_2 Batteries: A Kinetic Monte Carlo Approach. *J. Electrochem. Soc.* **2016**, *163*, A329–A337.

(2) Reuter, K. First-Principles Kinetic Monte Carlo Simulations for Heterogeneous Catalysis: Concepts, Status, and Frontiers. *Model. Simul. Heterog. Catal. React.* **2011**, 71–111.

(3) Bickel, T. A Note on Confined Diffusion. *Phys. A Stat. Mech. its Appl.* **2007**, *377*, 24–32.
